# Durable effect of imatinib and metronomic chemotherapy with capecitabine in pancreatic carcinoma

**DOI:** 10.3332/ecancer.2023.1535

**Published:** 2023-04-21

**Authors:** Sana Al-Sukhun, Karim Khalidi

**Affiliations:** 1 Al Hyatt Oncology Practice, 40 Ibn Khaldoon Street, Amman 11183, Jordan; 2 Radiology Department, Al Khalidi Hospital & Medical Centre, 40 Ibn Khaldoon Street, Amman 11183, Jordan

**Keywords:** pancreatic cancer, metronomic chemotherapy, molecular profiling, KIT amplification

## Abstract

**Background:**

Pancreatic ductal carcinoma (PDC) is a challenging diagnosis with a particularly poor prognosis, even after curative surgery (median survival: <30 months). The prognosis of borderline resectable pancreatic cancer (BR-PDC) is even worse. We describe a patient with BR-PDC who achieved stable disease with metronomic chemotherapy after refusing surgery.

**Case presentation:**

A 75-year-old woman was presented with jaundice and epigastric pain. Imaging confirmed a mass in the pancreatic head encasing the superior mesenteric vein, with obstruction of the pancreatic and bile ducts. After stenting to relieve the obstruction, Fine needle aspiration (FNA) confirmed the diagnosis of PDC. The patient refused surgery and radiation therapy but agreed for chemotherapy. After the second cycle of mFOLFIRINOX – complicated by febrile neutropenia – she refused further IV therapy. Genomic profiling revealed KIT amplification. Therefore, she was started on imatinib with dramatic improvement both clinically and biochemically reflected in carbohydrate antigen 19-9 drop. However, that response was short-lived at 3 months. Therefore, capecitabine was added at a low dose of 1 g bid on an alternate weekly basis. The patient did well and she is currently alive with a stable disease as of 2 years after diagnosis.

**Conclusion:**

Metronomic chemotherapy, especially capecitabine added to the targeted therapy, imatinib, is a potentially useful treatment for PDC where no other options are available, especially those harbouring no mutation in the dominant four genes. Indeed, the absence of mutation with KIT amplification could be a potential marker for improved outcomes with targeted and metronomic therapy, which deserves further evaluation in a clinical trial setting.

## Introduction

Pancreatic ductal carcinoma (PDC) is currently one of the leading causes of morbidity and mortality attributed to cancer worldwide. The rising burden is expected to move it to the second leading cause of worldwide cancer death by 2030 [1]. Most patients present with locally advanced or metastatic disease [2] where the mainstay of treatment is systemic chemotherapy [3]. Therefore, the prognosis remains poor with a median 5-year survival rate of just 10%, despite some improvement in therapeutic options [4]. Even those patients presenting with localised PDC who undergo surgery with or without adjuvant therapy will develop metastatic disease and rarely achieve a disease-free survival (DFS) of more than 2 years. Therefore, surgery alone is not sufficient for cure, and occult micrometastases are likely to be present at the time of diagnosis. These observations motivated the adoption of treatment sequencing, which includes neoadjuvant systemic therapy+/− radiation therapy and the application of surgery to the population of patients with stable or responding disease, most likely to receive clinical benefit from such large operations [5].

For patients who cannot tolerate or progress on conventional chemotherapy, very few options remain. Radiation therapy is associated with significant morbidity, especially among the elderly [6]. Advances in precision medicine for PDCs according to the genetics and molecular biology of this disease are promising approaches to overcome the heterogeneity of different patients and improve survival outcomes for this poorly prognostic disease. Only 25% harbour actionable molecular alterations, leaving the rest in need of other innovative approaches [7].

Conventional chemotherapy toxicity prompted the development of the concept of metronomic chemotherapy [8]. Metronomic therapy entails continuous administration of a drug, without long break-off therapy, but at lower doses than those used for conventional schedule [9]. The metronomic approach is convenient when the goal is to control disease while maintaining the quality of life in a fragile patient [10]. This approach is not yet the standard of care in any tumour despite its popularity; therefore, most aspects of this approach are determined by the physician according to the patient performance status and the tumour characteristics, including the choice of a chemotherapeutic agent, the dose and the schedule [11, 12].

Here, we report a case of localised pancreatic adenocarcinoma harbouring KIT amplification, demonstrating an exceptional response to initial standard chemotherapy, followed by stabilisation on imatinib and capecitabine maintenance.

## Case report

**Figure 1.**
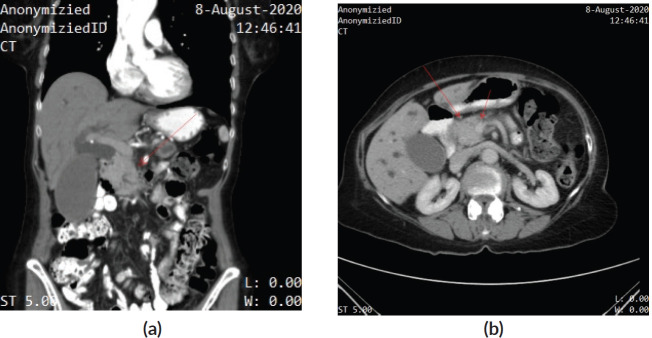
(a): Contrast-enhanced CT – coronal section – showing a mass at the head of the pancreas, encasing the superior mesenteric vein. (b): Contrast-enhanced CT showing a mass at the head of the pancreas, encasing the superior mesenteric vein.

A 75-year-old woman was hospitalised for management of obstructive jaundice and intermittent epigastric pain in August 2020. Contrast-enhanced computed tomography (CT) showed a mass at the head of the pancreas, encasing the superior mesenteric vein (Figure 1a and b). Positron emission tomography CT further delineated a mass in the uncinate process of the pancreas (size = 4.1 * 3.6 cm; SUV = 18.2) without a clear boundary with the duodenum. However, no distant metastases were detected. Endoscopic retrograde cholangiography revealed dilatation of the common bile duct, and an EBD tube was placed to drain the bile. The brush biopsy was unremarkable for malignancy. CT-guided fine needle aspiration biopsy of the pancreatic mass was concordant with adenocarcinoma, and the patient was diagnosed with potentially resectable PDC. The tumour marker, carbohydrate antigen 19-9 (CA19-9), was mildly elevated at 50.3 U/mL. After discussion, the multiple disciplinary teams recommended induction therapy followed by surgery, but she refused both surgery and radiation therapy and agreed only to chemotherapy.

Chemotherapy was commenced with modified FOLFIRINOX (leucovorin/5-FU/irinotecan/oxaliplatin). The first cycle was uneventful, but the second cycle was complicated by hospital admission for the management of diarrhoea, dehydration and urosepsis. The patient refused any further intravenous chemotherapy. She was willing, however, to try other oral options.

Comprehensive genomic profiling using liquid biopsy to perform next generation sequencing of the cell-free DNA (Guardant 360 CDX) was requested. The test detected KIT amplification at a plasma copy number of 2.2. The tumour was microsatellite stable.

Given the patient’s willingness to attempt new treatments that could provide her relief and improve her quality of life, treatment with KIT inhibitor was discussed and agreed to start, which had never been attempted in this population of patients. Imatinib, a tyrosine kinase inhibitor with activity against ABL, BCR-ABL, platelet derived growth factor receptors (PDGFRA), and c-KIT, was started on a 400-mg daily dosage [13]. Imatinib was chosen considering its ready availability and reasonable price as compared to other potential targeted therapeutics for c-KIT. In addition, it is a quite tolerable option. On a follow-up consultation a month after the start of treatment, the patient reported feeling well, her performance status improved to ECOG 0, and CA19-9 dropped to 7.

Three months after commencing maintenance imatinib (5 months after her initial diagnosis), imaging revealed an excellent ongoing response with stable disease. However, biochemical progression was evident in rising CA19-9 up to 117, possibly representing a resistant clonal population. Because she refused intravenous therapy, capecitabine was added to the ongoing imatinib therapy, at a reduced dose of 1 g bid × 1 week alternating with 1 week break off therapy. An alternate week schedule was used to improve tolerability as the patient developed GII/III ‘Hand-Foot syndrome’. Since imatinib was well tolerated, we continued imatinib with capecitabine.

**Figure 2.**
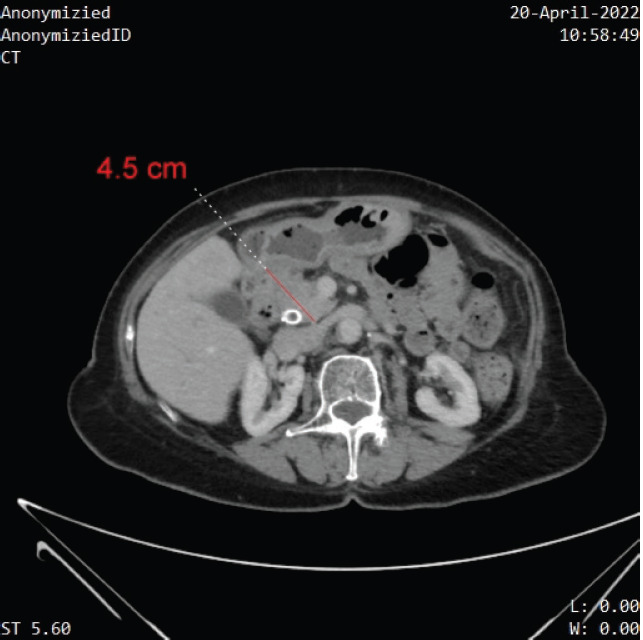
Contrast-enhanced CT demonstrating the stability of the mass at the head of the pancreas without further progression, or distant metastases.

Ongoing therapy with imatinib and capecitabine has been well tolerated, and she remains clinically well with excellent PS. CA19-9 dropped down to 11. Imaging at 16 months after the addition of capecitabine (20 months after her initial diagnosis) has revealed stable disease, with no metastatic disease on CT imaging (Figure 2).

The patient described in this report provided informed written consent for the collection and publication of her deidentified data – clinical, molecular and images – from her medical records.

## Discussion

Systemic therapy is the mainstay of treatment for PDC, an aggressive tumour with a typically advanced presentation [3]. Different regimens, tailored to the performance status of the patient, are recommended by current guidelines, but none is tailored to the underlying molecular alterations of the tumour [14]. Hence, the modest palliative effects of conventional chemotherapy, regardless of several cytotoxic drugs are combined. Usually, this therapy is associated with significant morbidity that negatively affects the quality of life. Even for those with potentially resectable disease, the likelihood of successful surgery after induction therapy for patients with resectable, borderline resectable, locally advanced type A, and locally advanced type B disease is approximately 90%, 75%, 60%, and 25%, respectively [5]. Our patient, therefore, made the decision not to undergo surgery and to get a treatment that does not impair her QoL.

Recently, it has been demonstrated that four frequently mutated genes: KRAS, TP53, CDKN2A (p16) and SMAD4 dominate the genetic landscape of PDC [15]. The number of mutations among the major four driver genes was substantially associated with OS and DFS [16]. The fewer mutations displayed by PDAC, the better the prognostic outcome in 71 patients who underwent a radical operation followed by adjuvant chemotherapy, with better OS predicted among those with zero to two mutated genes [17]. Our patient’s tumour displayed no mutation in any of those genes, consistent with the improved outcome. Response to imatinib was excellent, as predicted by its reported inhibitory effect in pancreatic cancer cell lines [18]. Overexpression of multiple tyrosine kinase growth factor receptors and their ligands has been described in pancreatic cancer cells, possibly supporting pancreatic cancer cell growth in a redundant manner. Imatinib inhibitory effect in pancreatic cancer cell lines did not impact growth factor-induced receptor and mitogen-activated protein kinase (MAPK) phosphorylation, possibly contributing to a short-lived response, as observed in our patient [18]. Repeat genomic testing was not attempted as it was too soon to repeat, within 3 months of therapy; especially since it was expensive and the patient herself was paying for her treatment. Similarly, mutational analysis was not requested due to the likelihood of low yield, in light of KIT amplification rather than a mutation in the original specimen.

5-FU has been the cornerstone of treatments for gastrointestinal cancer. There are a number of different treatment schedules in which it is used in different dose schemes without any definite differences in various non-inferiority studies [19], making the drug an ideal candidate to be used as metronomic chemotherapy. Another unique advantage is its antiangiogenic properties when administered continuously at low doses [18]. Therefore, we elected to start capecitabine as metronomic therapy to control the resistant clone/s. We took that approach in an attempt to reduce adverse events, including financial toxicity, and overcome resistance to conventional therapy [8]. Tolerability has been demonstrated in clinical trials [20]. Our patient did very well and was able to go on this schedule while maintaining an excellent quality of life. Metronomic treatment has previously been tested on breast cancer and prostate cancer, but the experience is less in the case of gastrointestinal cancer. Positive results have been demonstrated in colorectal cancer, oesophageal cancer, gastric cancer, biliary and liver cancer [10, 11, 21, 22].

## Conclusion

We report a locally advanced PDC patient who harbours KIT amplification with an exceptional response to targeted and metronomic therapy. Liquid biopsy was a great asset to offer our patient an option tailored to the particular tumour she has. It revealed the biology of the specific tumour under care, in light of the aforementioned molecular data [16]. Otherwise, she would have been on supportive care without any tumour-targeted therapy. The observed efficacy of this approach after first-line therapy draws attention to the importance of incorporating specific molecular characteristics of the tumour when making therapeutic decisions. More importantly, it emphasises the significance of addressing the heterogeneity of PDC when designing clinical trials as a means to overcome the poor prognosis of PDC and develop subtype-specific recommendations. Indeed, the absence of mutations with KIT amplification could possibly be a marker for improved outcomes with imatinib and metronomic therapy in patients with PDC. However, this is only one case with interesting clinical activity of the approach that merits further exploration in a clinical trial setting. In addition, it is a possible strategy when no other option is available.

## Conflicts of interest

None.

## Funding

No funding for this research was obtained from public or private sectors.
